# Defining the content of a minimal dataset for acquired brain injury using a Delphi procedure

**DOI:** 10.1186/s12955-020-01286-3

**Published:** 2020-02-17

**Authors:** Anne-Fleur Domensino, Ieke Winkens, Jolanda C. M. van Haastregt, Coen A. M. van Bennekom, Caroline M. van Heugten

**Affiliations:** 1 School for Mental Health and Neuroscience, Faculty of Health, Medicine and Life Sciences, Maastricht University Medical Center, Maastricht, The Netherlands; 2 Limburg Brain Injury Center, Maastricht, The Netherlands; 3 Department of Neuropsychology and Psychopharmacology, Faculty of Psychology and Neuroscience, Maastricht University, Maastricht, The Netherlands; 4 Care and Public Health Research Institute, Faculty of Health, Medicine and Life Sciences, Maastricht University Medical Center, Maastricht, The Netherlands; 5 Department of Research and Development, Heliomare Rehabilitation Center, Wijk aan Zee, The Netherlands; 6 Coronel Institute of Occupational Health, Amsterdam Public Health Research Institute, Academic Medical Center, University of Amsterdam, Amsterdam, The Netherlands

**Keywords:** Acquired brain injury, Minimal dataset, FAIR data, Delphi procedure, International Classification of Functioning, Disability and Health

## Abstract

**Background:**

The lack of consistency in outcome measurement within the field of acquired brain injury (ABI) leads to incomparability of collected data and, consequently, reduced generalisation of findings. We aim to develop a set of standardised measures which can be used to obtain the minimum amount of data necessary to characterise ABI-patients across all healthcare sectors and disciplines and in every stage of recovery; i.e., an ABI-specific minimal dataset (MDS-ABI). The current study was conducted to identify the core outcome domains for adults with ABI (what to measure?) and to select the most suitable measurements within these domains (how to measure it?).

**Methods:**

An initial comprehensive set of outcome domains and measurement instruments relevant for measuring the consequences of ABI was identified by a literature study. The selection of relevant domains was based on the International Classification of Functioning, Disability and Health framework. Measurement instruments were included in the Delphi procedure when they met pre-set requirements. A three-round Delphi study was conducted among Dutch experts (*n* = 48) using iterative web-based surveys to prioritise the proposed domains and instruments for the MDS-ABI. Throughout all rounds, participants could recommend additional or alternative domains and measurement instruments, and were fed back the collated group responses of the previous round.

**Results:**

Response rates ranged from 89 to 100%. After three rounds, the expert panel reached consensus (≥51%) on the inclusion of 12 outcome domains (demographics, injury characteristics, comorbidity, cognitive functioning, emotional functioning, energy, mobility, self-care, communication, participation, social support and quality of life), measured with six measurement instruments, two screening questions and a registry of demographic- and injury information. No consensus was reached on how to measure quality of life.

**Conclusions:**

The current study achieved consensus on the content of a minimal dataset for patients with ABI. The current version of the MDS-ABI will be evaluated and optimised if necessary in the near future.

## Background

Acquired brain injury (ABI) encompasses all types of damage to the brain that occur after birth and are not related to progressive diseases. As such, ABI typically results in a sudden decrease of functioning in various domains. Research into the sequelae of ABI in all of these domains leads to an abundance of data. These data, however, lack uniformity due to the broad spectrum of available measurement instruments, leading to incomparability of collected data and, consequently, reduced generalisation of findings [1]. Moreover, suboptimal coordination of instrument use could lead to unnecessary or duplicate data administration when patients receive care in different healthcare sectors or within multiple healthcare disciplines, leading to the need for patients to ‘repeat their story’. At the same time, the standardisation of outcome measurement could improve interdisciplinary communication and collaboration within healthcare for patients with ABI.

Recently, several studies have been conducted into the use of measurement tools in healthcare and research within the ABI population. For example, Tate et al. [2] systematically reviewed assessment tools used with adults in traumatic brain injury (TBI) research, identifying 728 unique instruments in several domains. Also, several systematic reviews into the measures of a particular domain have been conducted. For instance, Polinder et al. [3] identified 36 health-related quality of life measures used in TBI research, and recommended the establishment of consensus on methods of preference to facilitate comparability across studies. Furthermore, Tse et al. [4] reviewed participation measures used with stroke survivors and found 18 different measures, with none covering all aspects of participation as proposed by the International Classification of Functioning, Disability and Health (ICF) [5]. In conclusion, all studies revealed a high degree of variability in the use of measurement instruments, highlighting the need for consensus on preferred instruments for measuring outcome after ABI.

From a more general perspective, recent developments in the field of data collection promote findable, accessible, interoperable and reusable (FAIR) data, emphasizing the need for comparable data that can be integrated from non-cooperating resources with minimal effort [6]. Furthermore, international researchers, policymakers and clinicians have recognised the significance of FAIR data in healthcare by developing standardised outcome sets for specific populations or stages of a condition other than ABI [7–9].

Likewise, some initiatives have been undertaken for streamlining data collection in the field of ABI. One example is the National Institute of Health (NIH) recommendation to identify and use Common Data Elements with research in the field of neuroscience, such as the core dataset that was developed by Hicks et al. [10], aiding the standardised classification of injury severity after TBI. However, no Common Data Elements have been proposed for use with research into ABI in general. Similarly, the NIH recommends the use of PROMIS, a set of standardised generic patient report outcome measures [10, 11], comprehensive health-related quality of life measures specific for patients with neurologic conditions (NEURO-QOL) [12], and extensive standardised test batteries in several cognitive and emotional domains (NIH Toolbox) [13]. Another attempt at standardising data collection has been conducted by the International Consortium for Health Outcomes Measurement (ICHOM). The ICHOM initiative composes working groups of experts on specific health conditions to configure outcome sets that reflect what is most important to patients [14]. To date, one ICHOM set for stroke has been developed, but has yet to be evaluated [15]. Furthermore, the ICHOM sets tend to focus more on the neurological factors related to stroke. Hence, to date, there is no validated, compact dataset available for use in health care and research into all types and stages of ABI.

In the current project, we aim to develop a minimal dataset for adults with ABI (MDS-ABI). A minimal dataset is defined as a set of standardised measures used to index the minimum amount of data that is crucial for obtaining a global image of the patient across all healthcare sectors and disciplines and in every stage of the injury. It does not imply that outcome measurement needs to be restricted to the items that are in the set, as it can be expanded with measures of choice that serve a specific (research) goal [16]. The MDS-ABI aims to facilitate FAIR data collection on persons with ABI in the context of care, evaluation and research to improve the comparability of data, interdisciplinary communication and collaboration within the field of ABI, while reducing the burden of questionnaire administration for patients and healthcare professionals. This study was conducted to identify the core outcome domains for adults with ABI (what to measure?) and to select the most suitable measurements within these domains (how to measure it?).

## Methods

### Design

The current study comprised an online three-round Delphi procedure [17, 18] among experts on measurement instruments used in the field of ABI.

### Participants

Initially, potential experts were identified based on the professional network of the authors (criterion sampling). Invitees were then requested to propose supplementary potential experts within their own network (snowball sampling). Inclusion criteria were: being experienced in developing and/or evaluating measurement instruments that can be used to assess adults with ABI and being or having been employed in rehabilitation, neurology/neurosurgery, neuropsychiatry, elderly care, or disability care in the Netherlands.

### Procedure

Potential experts were asked by email to participate, before being admitted to the actual procedure. A briefing letter concerning the project, providing the outline and exact planning of the procedure was included with the e-mail. Potential participants were asked to judge whether they met the inclusion criteria and were willing to participate. Upon enrolment, participants provided information on their age, gender, occupation and experience with developing/evaluating measurement instruments. Participants received no compensation.

### Preparation phase

In the preparation phase, a literature study into outcome domains and measurement instruments relevant to measuring the consequences of ABI was conducted. First, identification of potentially relevant domains was done by the authors prior to consulting the experts and was based on the ICF model [5] and the ICF core sets for stroke [19] and traumatic brain injury [20]. Because we anticipated that some domains (such as mental functions) are more applicable to ABI than others, these domains were further divided into second-level categories (such as emotional functioning). Furthermore, since health-related quality of life is an important outcome measure in ABI research, but is not yet a confirmed domain in the ICF model [21], it was decided that this domain be proposed as a separate factor. Last, the ICF categories ‘activities’ and ‘participation’ were merged, since they have proven to be difficult to differentiate in outcome measurement [22]. A discussion among members of an advisory group consisting of persons with ABI and their informal caregivers (*n* = 17) confirmed the relevance of the domains that were identified by the researchers.

Second, we looked into measurement use in the field of ABI. In order to improve compatibility with other data collection initiatives and current clinical practice, the authors determined that the minimal dataset should be composed of existing measurement instruments. Accordingly, we made an inventory of measurement use in large studies and other data-shaping initiatives [15, 23–25] and benchmarks [26] in the field of ABI. Furthermore, the systematic review by Tate [2] and her compendium of tools used for measuring the outcomes of ABI [27] were consulted and served as a guide for classifying measurement instruments within ICF categories. Finally, evidence-based guidelines of several healthcare disciplines and sectors were checked for recommendations regarding the use of measurement instruments.

A list of requirements for analysing the suitability of measurement instruments for the MDS-ABI was composed through a survey study among members of the knowledge network that the current project is part of (*n* = 11). This network is comprised of Dutch healthcare professionals and researchers aiming to increase knowledge, cooperation and communication within the field of ABI. The survey study involved a digital form containing proposed requirements for measurement instruments in the MDS-ABI. Respondents reviewed the suggested requirements (necessary/preferable/unnecessary), leading to a set of requirements and preferences, as displayed in Table 1.

**Table 1:** Requirements and preferences to guide the inclusion of existing measurement instruments

Availability	Psychometric properties	Usability/feasibility
Requirements		
• The instrument must be available in Dutch. • The instrument must be in the public domain and thus freely available.	• The psychometric properties of the instrument must be known. • The instrument must not be diagnosis-specific.	• The instrument needs to be as short as possible. • There is as little overlap as possible with other measurement instruments.
Preferences		
• Preferably, the instrument is well-known and already frequently used in practice. • If possible, the instrument is frequently used in an international setting.	• The instrument is validated for Dutch adults with ABI.	• Preferably, all healthcare professionals should be able to administer the instrument; no specific training is required. • The materials required for administration are as few as possible.

All measurement instruments that were identified in the literature study were checked with our criteria and, when meeting all requirements, were entered into the first Delphi round.

### Delphi rounds

The executive phase of the study comprised a three-round Delphi procedure. Experts who agreed to participate received a personal invitation by e-mail containing an anonymised web link to the first Delphi round. In this round, participants were asked to indicate whether every proposed domain was important for the outcome measurement of persons with ABI, with three response options *(‘yes’, ‘no’ and ‘this is not my area of expertise’*). When this first question was answered affirmatively, the respondent was asked to indicate whether the measurement instruments proposed are suitable for the concerned domain (*‘yes’, ‘no’ and ‘no opinion’*). The first round was aimed at identifying potentially suitable measurement instruments for the MDS-ABI. Accordingly, respondents were not asked to express a preference for a particular measurement instrument until the second round, but rather were asked to indicate whether a proposed instrument would be suitable for the MDS-ABI.

The aspects that healthcare interventions focus most on, such as reducing symptoms, minimising disability and improving quality of life, can only be assessed by patient-reported outcome measures. Moreover, patient-reported outcome measures avoid observer bias and reduce the administrative burden of clinicians [28]. Therefore, respondents were stimulated to select patient-reported measurement instruments when appropriate. When respondents felt that a particular domain should be measured subjectively as well as objectively (by means of a test or observation measure), multiple instruments could be selected per domain in round one. Domains and instruments that reached a consensus of ‘no’ (≥51% of the respondents) were not taken into the next round.

Throughout all rounds, respondents were given the opportunity to elaborate on their decisions and to recommend additional or alternative domains and measurement instruments. Domains or instruments that were not in our list but proposed ≥ four times (i.e. > 10% of the sample) were included with the proposed domains and instruments in the next round. Measurement instruments that did not meet the requirements, (e.g. by not being freely available) were not presented in further rounds. In order to guide decision making on measurement instruments for the MDS-ABI, the requirements for measurement instruments and samples of the actual measures could be consulted using a hyperlink. Collective responses were fed back to the participants anonymously in the next round, using an information letter that was sent to the participants by e-mail. Participants who did not complete one of the three rounds were excluded from further participation.

The first Delphi round yielded consensus on the inclusion of all proposed outcome domains. Despite the fact that consequences of ABI can occur in all of these domains, measuring the full range of applicable ICF domains would be beyond the scope of a minimal dataset. Therefore, identification of the core domains for the MDS-ABI was carried through in round two. In order to narrow the selection of key domains, we sorted the domains that reached consensus in round one ascendingly by the percentage of ‘Yes’ answers, and asked respondents to reassess the proposed domains, keeping in mind that a minimal dataset needs to be compact and can be composed only of domains that are applicable to *all* adults with ABI.

Regarding domains for which no consensus was reached on the level of measurement instrument, participants were asked to indicate their preferences, by putting the proposed instruments in order of preference (*first place = most preferred, last is least preferred)*. For some domains (such as emotional functioning), the multiple instruments that were regarded as suitable for the MDS-ABI by respondents in the first round measured different constructs (behaviour and depression/anxiety, respectively). In these cases, respondents had to indicate whether they wanted both, one or none of the instruments to be included in the MDS-ABI. Domains and instruments for which consensus was reached were entered into the concept MDS-ABI.

For some domains, no suitable instrument was identified by the expert panel. Therefore, the use of a screening question was reviewed by the respondents in the third round. Moreover, the third Delphi round contained questions to help clarify the last issues regarding overlap between selected instruments.

### Analyses

The surveys were conducted using Qualtrics software [29]. All analyses were conducted using SPSS statistical software, version 24 [30]. Descriptive statistics were used for responder characteristics. Frequencies were calculated for ratings on multiple choice questions, excluding respondents who indicated they had no opinion on the particular subject. For ranking questions, mean ranks, standard deviation of the mean ranks, sum of ranks and the number of times the instrument was most preferred (first ranks) were calculated to indicate group preferences.

The level of agreement in order to reach consensus in Delphi procedures is not clearly defined [31]. The research team set this level a priori on the majority of respondents (51%) in the current study for multiple choice questions. Consensus for rating questions was defined as an item that has the lowest mean rank, combined with the highest number of first ranks. Any discrepancies between these two outcomes meant that consensus was not reached.

## Results

### Participants

**Fig. 1:**
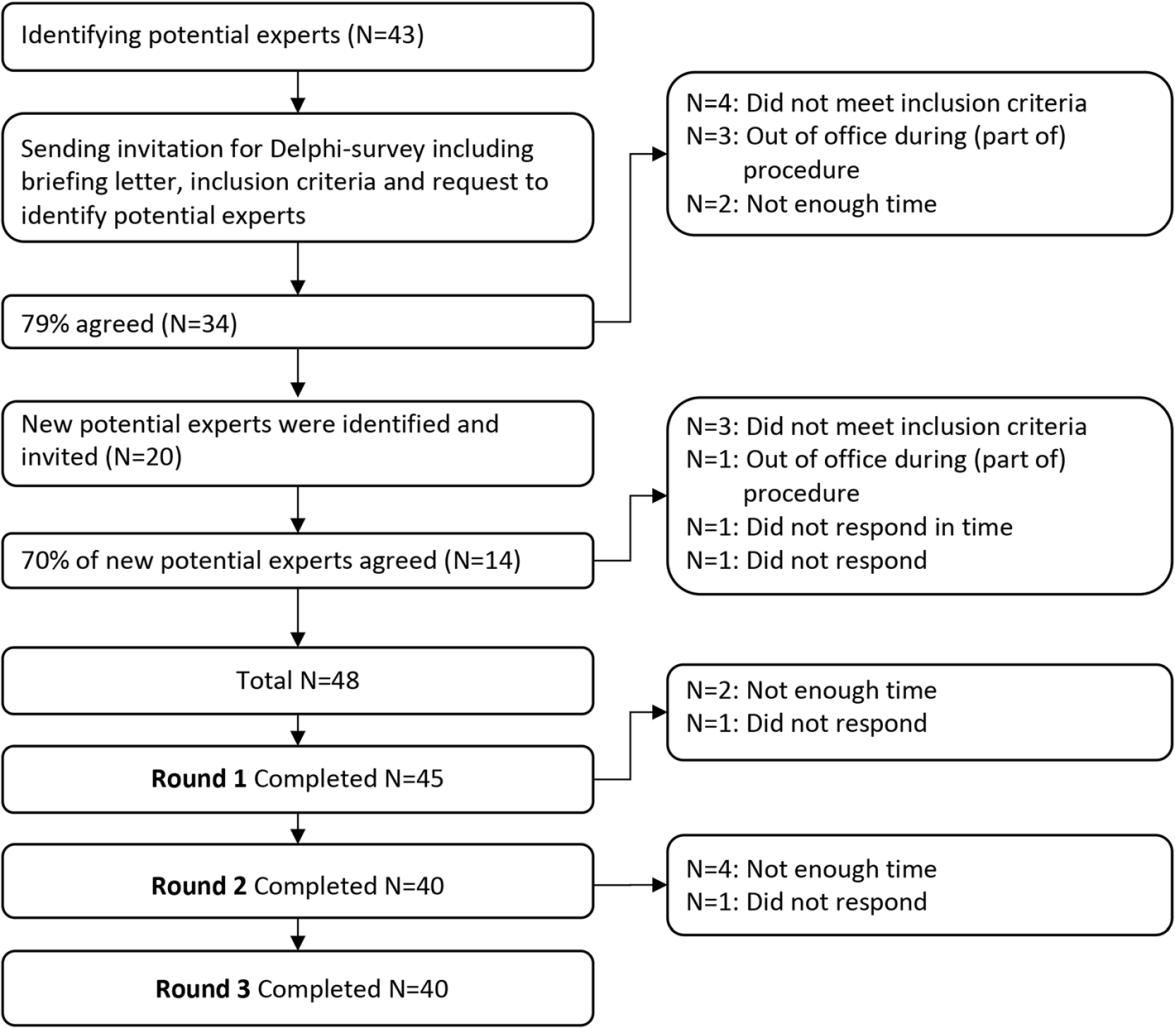
Flow of participants in Delphi procedure

Initially, we identified 43 potential experts, of which 34 (79%) agreed to participate. These participants identified another 20 potential experts. Of these newly identified potential experts, 14 (70%) agreed to participate, leading to the inclusion of 48 experts in total. Forty-five of the included experts responded to the first round (94% response rate). Forty of these 45 experts responded to round two (89%). These 40 experts were invited to round three, to which all (100%) responded (Fig. 1). Experts were employed in a diverse range of occupations and worked in several sectors of health care. Table 2 displays relevant participant characteristics.

**Table 2:** Characteristics of the expert panel (*n* = 45)

Characteristic	Mean ± sd / n(%)
Sex: males, *n* (%)	19 (42)
Age in years, mean ± (SD)	48.1 ± 11.5
Years of experience with developing/evaluating measurement instruments, mean ± (SD)	16 ± 11
Sector: *n* (%)^a^	
Elderly care	7 (9)
Rehabilitation	14 (18)
Mental health	9 (12)
Primary care Disability care	3 (4) 5 (6)
Hospital Higher education	17 (22) 18 (23)
Other	4 (5)
Occupation: *n* (%)	
Psychologist	18 (40)
Physician	12 (27)
Researcher	11 (24)
Other healthcare professionals	4 (9)

^a^Multiple entries were allowed

### Round 1

In the first round, all 18 proposed domains were selected with consensus rates ranging between 60.5–100%. For an overview of all domains and corresponding result, see table in Additional file 1. Seven additional domains were mentioned ≥ four times by the expert panel: voice and speech functions (*n* = 4), behaviour (*n* = 4), financial situation (*n* = 5), social network (*n* = 6), religion (*n* = 4), education (*n* = 6) and personality (*n* = 7). Because behaviour is not a separate category in the ICF model, but is covered by several other domains, such as ‘regulation of emotion’ within ‘emotional functioning’ [5] and instruments assessing behavioural problems were already proposed under ‘emotional functioning’, the domain ‘behaviour’ was not entered into subsequent rounds. Furthermore, although personality has proven to be an important predictor of outcomes after ABI [32], classifying it requires comprehensive testing and therefore it is not within the scope of the MDS-ABI. The remaining five domains were presented to the respondents in round two. Last, ≥ four participants noticed the overlap between ‘movement-related functions’ and ‘mobility’. Therefore, from this point, these domains were merged under ‘mobility’.

On instrument level, 35 of the instruments proposed by the authors were considered suitable for measurement of the corresponding domains. See table in Additional file 2 for the ratings per instrument. Immediate consensus on measurement level was achieved for the domains pain and comorbidity. Also, for these domains, no additional measurement instruments that met the requirements were proposed by the respondents.

No immediate consensus was reached for the remaining domains. In addition, in the first round, the experts proposed three new instruments that complied with the requirements and were therefore advanced into the second round. Two of these instruments were intended for measuring energy/fatigue; the Dutch Multifactor Fatigue Scale [33] and the Checklist Individual Strength [34]. The third item that was proposed reflected the use of a screening question rather than a full measurement instrument for measuring social support: “Does the patient have an informal caregiver?”

Finally, within the domain ‘injury characteristics’, the definition of ‘duration of hospital stay’ and ‘discharge destination’ needed to be further clarified, judging by the feedback of the respondents. Therefore, more detailed definitions (‘duration of hospital stay after acquiring the brain injury’ and ‘discharge destination from hospital stay’, respectively) were proposed to the respondents in round two.

### Round 2

In the second round, consensus was reached on a total of 12 domains; these make up the domains of the concept MDS-ABI (Additional file 1). For all of the selected domains, consensus on measurement level was reached (Additional file 3).

For the domains ‘communication’ and ‘support’, the majority of the respondents opted for the use of a screening question, leading the research team to formulate and propose a screening question for the domain ‘communication’ to the respondents in round three. The screening question that had been proposed by one of the participants in round one was proposed to the panel in round two. However, the results pointed to the need for rephrasing the screening question, as it was deemed inappropriate by four participants. Therefore, in round three, we asked whether respondents would prefer the screening question “Do you experience sufficient support from the people in your surroundings?” over “Does the patient have an informal caregiver?”.

Furthermore, there was some overlap between the instruments that were selected for mobility and self-care. The instrument that was selected to measure ‘mobility’ in the MDS-ABI, the Functional Ambulation Categories (FAC) [35], showed a clear resemblance to the instrument that was selected to measure ‘self-care’, the Barthel Index [36]. For instance, both instruments addressed the ability of ABI-patients to make transfers, to walk and to ascend stairs. Moreover, the satisfaction subscale of the Utrecht Scale for Evaluation of Participation – Rehabilitation (USER-P) [37] that was selected to measure ‘participation’, and the Life Satisfaction Questionnaire (LiSat) [38] that was favored to measure ‘quality of life’ showed significant overlap as well. Therefore, in the subsequent round, participants were asked to indicate which of these instruments should be selected for the concept MDS-ABI. Last, the majority of respondents agreed with the proposed definitions for ‘duration of hospital stay’ and ‘discharge destination’. These newly defined variables were taken into the next round so that respondents could judge their suitability.

### Round 3

On the question of how to settle the overlap between the measures that were selected for the domains ‘mobility’ and ‘self-care’, 83% of the respondents opted to drop the FAC and instead measure both domains with the Barthel Index. Also, both screening questions that were proposed to measure the ‘communication’ and ‘social support’ domains were agreed on by 90 and 82% of the respondents, respectively. Last, 62.5% opted for the inclusion of both newly defined variables ‘duration of hospital stay’ and ‘discharge destination’. On the contrary, no consensus was reached on how to solve the overlap between measures of participation and quality of care. For more information about the content of the third survey, please refer to the table in Additional file 4. Table 3 displays an overview of the first concept version of the MDS-ABI, drafted after the results of the current Delphi study.

**Table 3:** Domains and outcome measures that were selected by the Delphi expert panel

ICF chapter	Selected instrument or variable
Selected domain	
Disease characteristics	
Injury characteristics	Date of brain injury, type of brain injury, previous brain injury, duration of hospital stay, discharge destination.
Comorbidity	CIRS
Body functions & structure	
Cognitive functioning	MoCA
Emotional functioning	HADS
Energy	FSS
Activities and participation	
Mobility and Self-care	BI
Communication	*“Since acquiring brain injury, does the patient have trouble communicating (expressing and/or understanding)?”*
Participation	USER-P
Social support	*“Do you experience sufficient support from the people in your surroundings?”*
Personal factors	
Demographic characteristics	Age, sex, living situation, marital status, children.
Other	
Quality of life	No consensus

*CIRS* Cumulative Illness Rating Scale [39], *MoCA* Montreal Cognitive Assessment [40], *HADS* Hospital Anxiety and Despression Scale [41], *FSS* Fatigue Severity Scale [42], *BI* Barthel Index [36], *USER-P* Utrecht Scale for Evaluation of Rehabilitation – Participation [37]

## Discussion

The current study employed a Delphi procedure to define the content of a minimal dataset for outcome measurement in the field of ABI. The expert panel identified 12 domains of importance and reached consensus on how to measure 11 out of the 12 selected domains.

No consensus was reached on how to settle the overlap between the measurement instruments that were selected for the domains ‘participation’ and ‘quality of life’. Respondents showed a clear pattern of reasoning for their decisions: experts either wanted to limit the length of the MDS-ABI and thus chose the USER-P, or considered it important to include a validated measure of quality of life, leading them to choose the USER-P in combination with the 12-Item Short Form Health Survey (SF-12) [43]. Since this dichotomy reflects a matter of personal preference, we believe a supplementary Delphi round would have yielded similar results. Initially, we adopted the inclusion of both the USER-P and the SF-12 in the MDS-ABI, considering the fact that this option received the highest preference scores. However, after the first draft version of the MDS-ABI was established, it appeared that the SF-12 - although freely available - cannot be scored without the use of purchased software. Consequently, the answer with the second-highest preference scores, i.e. applying the USER-P for measuring both ‘participation’ and ‘quality of life’, was put into practice in constructing the concept MDS-ABI. As a result, the MDS-ABI does not strictly cover a measure of quality of life.

### Features of the draft MDS-ABI

Administration of the clinician-rated elements of the draft MDS-ABI will take approximately 25–40 min, based on the added average administration duration of all the measurement instruments. Completion time for the patient-reported questionnaires and screening questions is approximately 30 min. Because of its wide array of outcome domains and relatively limited administration length, the MDS-ABI could be used for screening purposes in health care to assist anamnesis, diagnosis and determination of appropriate treatment and to serve as an outcome measure in research contexts. The draft MDS-ABI contains existing measures that are available in Dutch as well as other languages; most of these measures are frequently used in international settings. All measurement instruments are of sufficient methodological quality and are accessible without restrictions. The measurement instruments and screening questions are not specific to conditions within the spectrum of ABI. Therefore, the MDS-ABI can facilitate quick, standardised data collection on all ABI-patients.

Since the current MDS-ABI consists of several patient-reported outcome measures, it is intended for use with adult patients who are capable of self-reporting on suitable domains. For ABI patients who are unable to complete these questionnaires, a so-called ‘proxy-module’ for all possible domains will be developed which can be administered by a professional or informal caregiver to retrieve essential information on injury outcomes. Likewise, since the MDS-ABI contains questionnaires that are irrelevant for patients in the acute phase of their injury, the authors recommend administering the HADS, FSS, and USER-P only in later stages of ABI.

Given that the current study was aimed at identifying important outcome domains for patients with ABI, no measurement instrument was included with regard to caregiver burden. Although ABI can induce a great and lasting burden on caregivers [44], identifying a suitable measure for the caregivers of persons with ABI was beyond the scope of this research. Future steps in the development of the MDS-ABI need to focus on incorporating caregiver experiences as well, especially in the proxy-module.

### Strengths and limitations

The use of a Delphi procedure is a particular strength of the current study. As a Delphi procedure usually is undertaken through multiple rounds of surveys, it allows respondents to adjust their opinion based on other participants’ knowledge, thereby transforming several personal opinions into a broad consensus [45]. Responses are anonymous; therefore, the Delphi procedure incorporates the advantages of group-based decision making without the disadvantages of face-to-face meetings such as practical implications and dominant characters [46].

The Delphi panel was composed of 45 healthcare professionals employed in a variety of sectors and disciplines of ABI health care. Accordingly, the results are considered to reflect the general opinion of experts across the field. In consulting experts rather than launching an established MDS-ABI, we anticipated stimulating bottom-up evolution of the dataset to improve support for the use of the MDS-ABI.

Given the fact that experts could access every proposed measurement instrument using survey hyperlinks, we estimate that respondents based their opinions on the quality and appropriateness of measurement instruments rather than familiarity alone.

The current study has several limitations. First, despite of the diversity of the healthcare sectors and disciplines represented by the Delphi panel, the sample was drawn from the professional network of the research team and consisted solely of Dutch participants; hence, it may not represent the opinion of all experts in the national and international field. Nonetheless, all selected measurement instruments are available in English and therefore, the MDS-ABI is suitable for international use. Nonwithstanding, we acknowledge that preferences for specific instruments may differ among countries. For instance, to date, the USER-P has mostly been used in Dutch studies.

Second, persons with ABI were not part of the actual Delphi procedure, although they defined crucial outcome domains during a group meeting preceding round one. ABI patients were not enrolled in the surveys because they lack expertise on measurement instruments and their psychometric properties. However, the experiences of persons with ABI will be evaluated in the consecutive feasibility study, in which the relevance of the selected domains and measures will be evaluated.

Moreover, some remarks should be made on the selected measurement instruments in the MDS-ABI. As the aim of a minimal dataset is to serve as a screening instrument of confined length, the selection of measurement instruments was limited to instruments with a short administration duration. A comprehensive assessment of certain domains arguably offers a better understanding of complaints than could be obtained by using shorter screening instruments. Similarly, there are widely used instruments of high quality that have been developed for patients with a specific diagnosis. Although the inclusion of such measures would not be feasible for a minimal dataset aimed at the heterogeneous group of people with ABI, comprehensive measures such as personality questionnaires, and measures designed for specific diagnoses such as scales assessing arm functioning after stroke, can be added to the MDS-ABI to preference.

Furthermore, only those instruments that were validated and translated into Dutch at the time of the first round of the Delphi procedure complied with the requirements and were proposed to the participants. As a result, recently developed measures were not considered. As outcome measurement after ABI is a developing field, the MDS-ABI will need to be updated regularly in the future.

Last, no suitable instrument was identified for measuring the domains ‘communication’ and ‘social support’. Since both domains were marked as essential for the MDS-ABI, respondents opted to use screening questions. As no validated screening questions for these domains exist, these items were composed by the Delphi panel. Therefore, their sensitivity and feasibility need to be evaluated in future research.

### Future research

As a next step, we aim to evaluate the feasibility, usefulness and relevance of the MDS-ABI with healthcare professionals and with ABI patients in several sectors of Dutch health care. On the long term, the MDS-ABI needs to be updated according to future developments in the field of minimal data collection, such as the development and implementation of comprehensive item banks. Such item bank systems, for example PROMIS [11], can be administered more efficiently using Computerized Adaptive Testing (CAT) [47]. However, this application is still under construction for most domains and therefore currently unavailable for use as part of the MDS-ABI. Once fully operative, CAT-administered item banks of the self-reported domains could be integrated into the MDS-ABI. Nonetheless, we value the additional clinician-rated parts of the MDS-ABI to obtain a complete image of the patient’s status.

## Conclusions

By performing a three-round Delphi study, the current study achieved consensus on the content of an ABI-specific minimal dataset. The expert panel selected twelve outcome domains (demographics, injury characteristics, comorbidity, cognitive functioning, emotional functioning, energy, mobility, self-care, communication, participation, social support and quality of life), measured with six existing measurement instruments (CIRS, MoCA, HADS, FSS, BI, USER-P), two screening questions, and registry of demographic and injury information. No consensus was reached on how to measure quality of life.

By developing an ABI-specific minimal dataset, we aim to facilitate uniform data collection to increase comparability, promote data pooling, and improve communication in the field of ABI. Furthermore, we hope to relieve the administrative burden for both patients and clinicians. The draft version of the MDS-ABI that is under development is based on the outcomes of the Delphi procedure and will soon be further evaluated and adjusted if necessary.

## Supplementary information

**Additional file 1.** Results of voting for domains to be included in the MDS-ABI. Results are displayed on a dichotomous scale, excluding ‘this is not my area of expertise’. Domains that that reached consensus in the second round were included in the draft MDS-NAH.

**Additional file 2.** Results of voting for suitable measurement instruments per domain in round 1. Results are displayed on a dichotomous scale, excluding respondents who indicated ‘no opinion’.

**Additional file 3.** Responses (percentages) on questions about suitable measurement instruments per domain in round two. Table reflects domains for which multiple instruments measuring different constructs were selected in the previous round.

**Additional file 4.** Percentages of ‘yes’ on response categories of questions regarding the design of the concept MDS-ABI in round three.

## Data Availability

All data generated or analysed during this study are included in this published article and its supplementary information files.

## Abbreviations

ABI: Acquired Brain Injury
BI: Barthel Index
CIRS: Cumulative Illness Rating Scale
FAIR: Findeable, Accesible, Interoperable and Reusable
FSS: Fatigue Severity Scale
HADS: Hospital Anxiety and Despression Scale
ICF: International Classification of Functioning, Disability and Health
ICHOM: International Consortium for Health Outcomes Measures
LiSat: Life Satisfaction Questionnaire
MDS-ABI: Minimal Dataset for adults with ABI
MoCA: Montreal Cognitive Assessment
NIH: National Institute of Health
SF-12: 12-Item Short Form Health Survey
TBI: Traumatic Brain Injury
USER-P: Utrecht Scale for Evaluation of Rehabilitation – Participation
