# Supplementary material for: Defining the content of a minimal dataset for acquired brain injury using a Delphi procedure
Source: Health Qual Life Outcomes. 2020 Feb 17;18:30. doi: 10.1186/s12955-020-01286-3 (PMC7027079; doi:10.1186/s12955-020-01286-3)
Supplement: Supplementary file 1 — Additional file 1. Results of voting for domains to be included in the MDS-ABI. Results are displayed on a dichotomous scale, excluding ‘this is not my area of expertise’. Domains that that reached consensus in the second round were included in the draft MDS-NAH. [file 12955_2020_1286_MOESM1_ESM.pdf]

Domains	Yes (%)	
	Round 1	Round 2
**Disease characteristics**		
Injury characteristics	95.3*	82.5*
Comorbidity	90.9*	75.0*
**Body functions and structures**		
Cognitive functioning	100.0*	97.5*
Emotional functioning	97.7*	87.5*
Energy	86.4*	72.5*
Sensory functioning	66.7*	27.5
Pain	60.5*	27.5
Voice and Speech[a]	-	17.5
Movement-related functions[b]	91.2*	-
**Activities and participation**		
Participation (including domestic life, civil life and major life areas)	97.6*	90.0*
Communication	88.4*	87.5*
Mobility	91.7*	77.5*
Self-care	90.7*	75.0*
**External factors**		
Products and technology	70.6*	25.0
Services, systems and policies	71.1*	32.5
Support	90.5*	75.0*
Social network	NP	42.5
**Personal factors**		
Demographic factors	90.5*	82.5*
Coping style	78.6*	47.5
Education[a]	-	45.0
Religion[a]	-	20.0
**Other**		
Quality of life	90.9*	77.5*

Note. *=reached consensus, [a]=proposed by respondents in the first round, [b]=merged with 'mobility' after first round.
