# Supplementary material for: Defining the content of a minimal dataset for acquired brain injury using a Delphi procedure
Source: Health Qual Life Outcomes. 2020 Feb 17;18:30. doi: 10.1186/s12955-020-01286-3 (PMC7027079; doi:10.1186/s12955-020-01286-3)
Supplement: Supplementary file 2 — Additional file 2. Results of voting for suitable measurement instruments per domain in round 1. Results are displayed on a dichotomous scale, excluding respondents who indicated ‘no opinion’. [file 12955_2020_1286_MOESM2_ESM.pdf]

Measurement instruments	Yes (%)	Measurement instruments	Yes (%)	Measurement instruments	Yes (%)	Measurement instruments	Yes (%)	Measurement instruments	Yes (%)	Measurement instruments	Yes (%)
**Disease characteristics**		**Body functions and structures**		**Activities and participation**		**Environmental factors**		**Personal factors**		**Other**	
**Injury characteristics**		**Cognitive functioning**		**Mobility**		**Social support**		**Demographics**		**Quality of life**	
Date of brain injury	91.1*	MOCA	89.7*	FAC	85.7*	MSPSS	76.9*	Age	82.2*	LiSat-9	81.3*
Type of brain injury	88.9*	CFQ	55.2*	MI	81.3*	SSL	57.9*	Sex	82.2*	SF-12	77.8*
Previous brain injury	86.7*	**Emotional functioning**		BBS	72.2*	**Services, systems and policies**		Living situation	80.0*	EQ-5D	75.0*
Discharge destination	64.4*	HADS	82.2*	10MWT	68.4*	General practitioner	48.9	Marital status	60.0*	WHOQOL-BREF	75.0*
Duration of hospital stay	53.3*	NPI-Q	69.7*	TGUG	66.7*	Medication	48.9	Children	53.3*	SF-36	69.2*
GCS	48.9	**Energy**		**Communication**		Physical therapist	42.2	Socioeconomic status	44.4	SWLS	50.0
ICD-10	40.0	FSS	83.9*	Instrument query	40.5	Psychologist	42.2	Ethnicity	40.0		
RLAS	13.3	BFI	41.2	**Participation**		Home care	42.2	**Coping**			
**Comorbidity**		**Pain**		USER-P	82.8*	Speech therapist	40.0	UPCC	55.2*		
CIRS	76.2**	VAS	95.0**	CIQ	72.2*	Occupational therapist	37.8				
LMV	46.7	**Sensory functioning**		FAI	63.2*	Hospital admittance	37.8				
CCI	30.8	Instrument query	38.5	IPA	41.2	Rehabilitation care	37.8				
				**Self-care**		Medical officer	33.3				
				BI	87.5*	Outpatient hospital care	31.1				
				KIADL	60.0*	Living facilities	31.1				
				mRS	45.5	Social work	28.9				

	IPA	33.3	First aid	24.4
			Dietician	17.8
			Homoeopathist	11.1
			**Products and technology**	
			Mobility aids	48.9
			Walking aids	48.9
			Communication aids	37.8
			Hearing aid	31.1
			Braces	28.9
			Glasses	26.7
			Bathroom aids	22.2

Note. GCS; Glasgow Coma Scale, ICD-10; International Classification of Diseases -10, RLAS; Ranchos Los Amigos Scale, CIRS; Cumulative Illness Rating Scale, LMV; Landelijke Monitor Volksgezondheid, CCI; Charlson Comorbidity Index, MOCA; Montreal Cognitive Assessment, CFQ; Cognitive Failure Questionnaire, HADS; Hospital Anxiety and Depression scale, NPI-Q; Neuropsychiatric Inventory-Questionnaire, FSS; Fatigue Severity Scale, BFI; Brief Fatigue Inventory, VAS; Visual Analogue Scale, FAC; Functional Ambulation Categories, MI; Motricity Index, BBS; Berg Balance Scale, 10MWT; 10 Minute Walk Test, TGUG; Timed Get Up and Go, USER-P; Utrecht Scale for Evaluation of Rehabilitation-Participation, CIQ; Community Integration Questionnaire, FAI; Frenchay Activity Index, IPA; Impact on Participation and Autonomy, BI; Barthel Index, KIADL; Katz Index of Activities of Daily Living, mRS; modified Rankin Scale, MSPSS; Multidimensional Scale of Perceived Social Support, SSL; Social Support List, UPCC; Utrecht Proactive Coping Competence scale, LiSat; Life Satisfaction Questionnaire, SF-12; 12-Item Short Form Health Survey, EQ-5D; EuroQol-5 dimensions, WHOQOL-BREF; World Health Organization Quality of Life Questionnaire-BREF, SF-36; 36-Item Short Form Health Survey, SWLS; Satisfaction With Life Scale. * = Rated as a suitable instrument for the concerned domain by the majority of respondents. ** = Only instrument for the concerned domain that reached consensus.
