# Supplementary material for: Defining the content of a minimal dataset for acquired brain injury using a Delphi procedure
Source: Health Qual Life Outcomes. 2020 Feb 17;18:30. doi: 10.1186/s12955-020-01286-3 (PMC7027079; doi:10.1186/s12955-020-01286-3)
Supplement: Supplementary file 3 — Additional file 3. Responses (percentages) on questions about suitable measurement instruments per domain in round two. Table reflects domains for which multiple instruments measuring different constructs were selected in the previous round. [file 12955_2020_1286_MOESM3_ESM.pdf]

Domain	Yes (%)
**Cognitive functioning**	
MoCa	68.6*
CFQ	2.9
Both	28.6
**Emotional functioning**	
HADS	57.6*
NPI-Q	15.2
Both	27.3
**Injury characteristics**	
**Do you agree with the definitions for 'duration of hospital stay' and 'discharge destination'?**	
Yes	90.6*
No	9.4
**Communication**	
**There is no instrument that met the preconditions. Should this domain still be included in the MDS-ABI?**	
Yes, with a screening question	85.7*
Yes and I know a suitable instrument:	8.6
No	5.7

Note. Montreal Cognitive Assessment, CFQ; Cognitive Failure Questionnaire, HADS; Hospital Anxiety and Depression scale, NPI-Q; Neuropsychiatric Inventory-Questionnaire. ** = Reached consensus.*
