# Supplementary material for: Defining the content of a minimal dataset for acquired brain injury using a Delphi procedure
Source: Health Qual Life Outcomes. 2020 Feb 17;18:30. doi: 10.1186/s12955-020-01286-3 (PMC7027079; doi:10.1186/s12955-020-01286-3)
Supplement: Supplementary file 4 — Additional file 4. Percentages of ‘yes’ on response categories of questions regarding the design of the concept MDS-ABI in round three. [file 12955_2020_1286_MOESM4_ESM.pdf]

Question	Yes (%)
**What variable should be included?**	
Duration of hospital stay	5
Discharge destination	22.5
Both	62.5*
None	10
**Do you agree with the proposed screening question for communication?**	
Yes	89.5*
No	10.5
**Should we drop the FAC in order to bypass the overlap between mobility and self-care?**	
Yes	82.8*
No	17.2
**Do you prefer the newly proposed screening question for social support over the question that was proposed by a respondent in round 2?**	
Yes	82.4*
No	17.6
**The content of the measurement instruments that were selected for participation and QoL overlap. What measures should be included in the MDS-ABI?**	
USER-P for participation and SF-12 for QoL	48.4
USER-P for both participation and QoL	38.7
USER-P for participation and LiSat for QoL	12.9

Note. USER-P; Utrecht Scale for Evaluation of Rehabilitation-Participation, LiSat; Life Satisfaction Questionnaire, SF-12; 12-Item Short Form Health Survey, QoL; Quality of Life. ** = Reached consensus.*
